# Changes in the Hormonal Profile of Athletes following a Combat Sports Performance

**DOI:** 10.1155/2020/9684792

**Published:** 2020-10-15

**Authors:** Andrzej Ziemba, Jakub G. Adamczyk, Anna Barczak, Dariusz Boguszewski, Agnieszka Kozacz, Jan Dąbrowski, Marta Steczkowska, Beata Pepłońska, Cezary Żekanowski

**Affiliations:** ^1^Department of Applied Physiology, Mossakowski Medical Research Centre, Polish Academy of Sciences, Warsaw, Poland; ^2^Department of Sport Theory, Józef Piłsudski University of Physical Education, Warsaw, Poland; ^3^Department of Rehabilitation, Physiotherapy Division, Medical University of Warsaw, Warsaw, Poland; ^4^Laboratory of Neurogenetics, Mossakowski Medical Research Centre, Polish Academy of Sciences, Warsaw, Poland

## Abstract

**Results:**

Following fighting, the adrenaline concentration was significantly higher in all athletes, most markedly in K (*p* < 0.001). Baseline cortisol and BDNF levels did not differ among the groups and rose significantly in all the groups after the performance. Baseline testosterone concentration was slightly higher in K than in JSW and rose in all the groups to reach similar levels; the increase in T was significantly higher than in K.

**Conclusions:**

Despite substantial differences in the characteristics of the combat sports investigated, including the type of physical effort and the required balance between restraint and aggression, the performance in each of them gives rise to similar hormonal changes with a possible exception of karate showing higher stress hormone levels.

## 1. Introduction

Stimulation of the sympathetic nervous system and the hypothalamic-pituitary-adrenal axis are among the main mechanisms of adaptation to physical exercise. The stimulation leads to, respectively, increased secretion of adrenaline and noradrenaline and enhanced production of glucocorticoids (cortisol). The activation results in a mobilization of energy reserves whose extent depends on the type of exercise [1]. It should be pointed out, however, that a similar hormonal response to that caused by physical activity is also evoked by stressful stimuli, including emotions. In combat sports, one finds an overlap of both factors, exercise and fight-related emotions/aggression. Since aggression comprises aggressiveness and anger (anger manifests in feeling hurt by a deliberate action against us, and aggressiveness is a readiness to respond to a situation with aggression [2]), some studies point to the fact that the level of sport-related aggressiveness is not discipline-specific but rather that individuals with higher aggressiveness may be naturally attracted to combat-type sports [3].

Combat sports are characterized by relatively short duration ranging from a few seconds to several minutes when the interval exercise involves large muscle groups performing both static and dynamic contractions. Additional specific features of combat sports include a direct physical contact with the opponent, which triggers psychological load and aggression. All these factors stimulate the response of the sympathetic system and the hypothalamic-pituitary-adrenal axis, and its intensity can be affected by the diversity of rules and styles of individual disciplines.

Although all combat sport disciplines have a common origin, they remain different with regard to the rules of the fight, its dynamics, and requirements posed on the athletes. For example, boxers and kick boxers must follow several rounds of high intensity effort with VO_2max_ potential approaching 65 ml/kg/min and blood lactate levels exceeding 13.5 ± 2 mmol/l [4]. On the other hand, taekwondo athletes use mainly leg techniques (kicking) [5] requiring relatively low maximal oxygen consumption (ca. 55 ml/kg/min) and inducing less-extreme lactate level (≈11 mmol/l) after fight [6]. At the other extreme are judo and wrestling of very high intensity (blood lactate after fight up to 20 mmol/l) requiring adequate blood buffering capacity that has to be developed in rigorous training [7]. At the same time, a highly efficient aerobic pathway seems to be crucial for repeated high-intensity interval efforts [6]. Also, judo, sumo, and wrestling involve ample static (armlocks, grappling techniques) and dynamic (throws) muscular work putting higher strength demands on the competitors than do karate or taekwondo [8]. In turn, from the point of view of psychological and hormonal requirements, a direct or indirect contact with the opponent and the risk of injury caused by the fight can be a decisive factor in training and competition requirements [9].

Considering the above-mentioned aspects, the aim of this study was to assess the changes of the hypothalamic-pituitary-adrenal axis hormone levels following performance in selected combat sports. In addition, in view of the positive impact of physical exercise (especially endurance exercise) on brain function and energy metabolism, we decided to determine BDNF concentration changes as well. This brain-derived neurotrophic factor is involved in memory and cognitive development, and its expression is increased by exercise [10].

## 2. Material and Methods

### 2.1. Participants

The study included 130 men practicing combat sports at a high level. The majority were Poland representatives in international competitions, including Olympic Games participants (*n* = 4) and World and European championships medalists (*n* = 44), and the rest of the study group represented a top national level (at least one medal won in national championships). Basing on the energy demands of individual discipline, their technical characteristics, and the degree and type of contact with the opponent during fight [6, 9, 11–13], the participants were divided into three groups: karate (K), taekwondo (T), and a combination of judo, wrestling, and sumo (JWS) (Table 1).  Table 2 shows basic characteristics of the study subjects and their training experience.

In accordance with the Declaration of Helsinki, the participants signed an informed consent approved by the Local Ethics Committee.

### 2.2. Procedures

Blood samples were taken from the antecubital vein prior to the fight and as soon as possible after its completion and stored at -80°C until analysis.

### 2.3. Analytical Methods

Adrenaline and noradrenaline were quantified by a radioimmunological method with commercial tests 2-CAT RIA (Demeditec Diagnostics GmbH, Germany) using a 1470 WIZARD Gamma Counter (PerkinElmer, Finland). Testosterone, cortisol, and BDNF were determined using specific ELISA kits from Demeditec Diagnostics GmbH (testosterone and cortisol) and R&D System, Inc. The assays were read in a Tecan SPARK 10M microplate reader (Tecan Austria GmbH) with SparkControl Magellan reader control and data reduction software.

### 2.4. Statistical Analysis

The Shapiro-Wilk test was used to assess the normality of variables. Because of a nonnormal distribution of values, the results before and after the fight were compared by the Wilcoxon test. Differences between disciplines were assessed by Mann and Whitney *U* test. *p* value below 0.05 was considered to be statistically significant. Statistical analyses were made using Statistica 10 (StatSoft, Tulsa, USA).

## 3. Results

Table 3 shows blood catecholamines, cortisol, testosterone, and BDNF levels in three groups: athletes involved in karate (K group) and taekwondo (T group) and in wrestling, judo, and sumo (JWS group).

Prior to the fight, a blood adrenaline level was similar in all groups (2.7 ± 0.2 ng/ml, 2.2 ± 0.3 ng/ml and 2.7 ± 0.3 ng/ml, respectively) (*p* > 0.05). After the fight the adrenaline concentration in all the athletes rose significantly (*p* < 0.0001) to 6.3 ± 0.7 ng/ml (group K), 3.8 ± 0.4 ng/ml (group T) and 4.9 ± 0.5 ng/ml (group JWS). The greatest increase in adrenaline concentration following the fight was observed in the karate group (Δ = 3.6 ± 0.7) whereas in taekwondo athletes the adrenaline increase was the smallest (Δ = 1.6 ± 0.24) (Table 3). The difference in the adrenaline concentration increase between the two groups was statistically significant (*p* < 0.02).

The resting blood noradrenaline level in the K group was 17.4 ± 1.9 ng/ml and 15.2 ± 0.9 ng/ml in the JWS group (*p* > 0.05) and significantly lower in the T group (9.5 ± 0.8 ng/ml, *p* < 0.001). Postfight, the level of noradrenaline increased significantly (*p* < 0.001) in all groups to reach 36.5 ± 4.8 ng/ml in the K group, 14.6 ± 1.3 ng/ml in the T group, and 23.6 ± 1.5 ng/ml in the JWS group. As indicated in Table 3, the highest increase of noradrenaline (the value of Δ) was recorded following a karate fight (Δ = 19.1 ± 3.6 ng/ml). It was significantly higher than those in the JWS group (8.4 ± 1.2 ng/ml, *p* < 0.001) and T group (Δ = 5.0 ± 0.7 ng/ml, which was significantly different from values obtained during karate fights, *p* < 0.001).

The blood cortisol concentration before the fight was 114.5 ± 16.2 ng/ml in the K group, and it rose significantly to 209.1 ± 24.2 ng/ml (*p* < 0.0001, Δ = 94.6 ± 20.2 ng/ml). Similar changes were identified in the T group where the cortisol level increased substantially after the fight from 106.3 ± 10.9 ng/ml to 202.4 ± 19.8 ng/ml (*p* < 0.001) and in the JWS group (from 90 ± 5.3 ng/ml to 152.4 ± 6.5 ng/ml, *p* < 0.001). The baseline cortisol levels did not differ significantly among the study groups whereas the lowest Δ value found in the JWS group (Δ = 61.2 ± 5.2) was statistically significantly different from the values in the two other groups (*p* < 0.001).

The prefight blood testosterone level was 4.3 ± 0.4 ng/ml in the karate group which was significantly higher than in groups T and JWS (3.7 ± 0.5 ng/ml, and 3.2 ± 0.2 ng/ml, respectively, *p* < 0.001). Following fight, the testosterone level increased significantly in all groups: to 5.6 ± 0.5 ng/ml (*p* < 0.001) in the K group, to 4.5 ± 0.5 ng/ml (*p* < 0.001) in the T group, and to 4.6 ± 0.2 ng/ml in the JWS group (*p* < 0.001). The lowest Δ value was observed in the taekwondo group (0.8 ± 0.13 ng/ml), than that in the K group (1.3 ± 0.16 ng/ml) and the JWS group (1.4 ± 0.2 ng/ml). The latter numbers were not significantly different (*p* > 0.05).

The basic blood BDNF level was similar in all the groups and amounted to 217.1 ± 24.1 pg/ml (K group), 231.4 ± 23.6 pg/ml (T group) and 228.0 ± 31.5 pg/ml (JWS group). After the fight a marked increase in BDNF level was observed in all the groups to reach the following values: 431.3 ± 63.8 pg/ml (Δ = 214.2 ± 71.4 pg/ml) in K group, 417.0 ± 56.8 pg/ml (Δ = 185.6 ± 84.3 pg/ml) in T group and 453.2 ± 86.2 pg/ml (Δ = 225.2 ± 79.4 pg/ml) in JSW group. Δ BDNF between the groups after the fight was statistically insignificant (*p* > 0.005).

The resting testosterone/cortisol ratio was similar in all groups (K:0.04 ± 0.01, T:0.03 ± 0.02, and JSW: 0.035 ± 0.02, *p* < 0.05) (Table 4). After performance in all the groups, the testosterone/cortisol ratio decreased significantly (*p* < 0.01), but there were no significant differences between the groups.

## 4. Discussion

This study showed a substantial increase in the blood adrenaline, noradrenaline, cortisol, testosterone, and BDNF levels following combat sports competition, consistent with earlier studies. These increases indicate a significant stimulation of the hypothalamic-pituitary-adrenal axis during physical effort.

To determine how the discipline-specific balance between aggression and composure affects the hormonal response, the athletes analyzed in this study were divided into three groups according to the combat technique and strategy, which have a great impact on the anger and aggression control [14]. Most karate and taekwondo competition involves predesigned attack, defense, and counterattack (*kata*) demonstration forms. The athletes are expected to perform a range of postures/poses and techniques attesting to their skills and versatility. Some kata techniques are meant to demonstrate speed; others emphasize special breathing techniques. The available literature reports that Olympic-level combat athletes generally show higher aggression indices than those performing at a lower level [14]. Furthermore, some karate variations involve only light-contact or semicontact fight where the competitors must precisely control the techniques they use. There is also the *Kano paradox*, stating that less-offensive techniques are better practiced during training are far more efficient and effective in a real fight that are the more offensive (potentially more dangerous) ones. So, in sports which involve kata and faking techniques, the athletes show lower preparedness to a real physical struggle with an opponent than do athletes in other combat sport disciplines [15]. This is a strong argument in favor of the discipline grouping used in the present study.

The requirements for sports with marking techniques, with karate and partly taekwondo being examples, are different than for a real physical contact fight (e.g., judo, kick-boxing, and wrestling). In such sports, what matters more than success in a competition is discipline, educational effect, physical fitness, and self-confidence.

Detailed characteristics of the physiological indices of judo competitors have been presented by Franchini et al. [12], who estimated a typical maximal oxygen uptake (VO_2max_) at approx. 50-55 ml/kg/min. The aerobic capacity of karate fighters has been reported to range between 47.8 ± 4.4 and 61.4 ± 2.6 ml/kg/min [16] and for taekwondo fighters -44 – 63 ml/kg/min [6]. These data provide sufficient ground to conclude that there are no fundamental differences in the physical capacity among athletes from various combat sports. The type of exercise in numerous combat sports involves short intervals (short breaks between attacks). Irrespective of the break-to-attack ratio, the energy required comes mainly from anaerobic metabolism which involves phosphocreatine (PCr) degradation and anaerobic glycolysis. Despite the leading role of the anaerobic processes, a high physical capacity of the athletes is essential for exhaustion prevention during both training and the rest between consecutive attacks as well as for rapid and effective after-fight recovery. In light of the results obtained, the hormonal axis analyzed here has been proven to be as important in the metabolic response to exercise as it is in endurance sports with the dynamic component prevailing.

It should be emphasized that until present no data were available indicating different physiological indices for specific combat sports; on the contrary, the papers cited above reported similarities in the aerobic capacity. Also other, indices show similar characteristics in different disciplines; for example, the body fat content is similarly low in karate, judo, and taekwondo fighters, except for the highest weight categories [1].

In contrast, our study found significantly higher catecholamine levels both before and especially after the fight in the karate group compared to the other combat sport groups.

A similar pattern was observed for testosterone, a hormone of a potent androgenic and anabolic activity, whose increase following fight was the highest in the karate group, significantly higher than in the taekwondo athletes. Also, earlier studies on diverse sport disciplines have noted substantial elevation of blood testosterone, especially in short-term and high-intensity sports. It could be due to an increased level of catecholamines and/or stimulation of the sympathetic system [17]. This would explain the observed similarities between the catecholamine and testosterone profiles of the individual study groups. Why the noradrenaline and testosterone levels increase significantly less in taekwondo athletes than in the karate group is not obvious. One possible explanation could be that the elite taekwondo athletes present the so-called iceberg profile typical of high-level sportsmen [14]. Also, the relatively low strength component in taekwondo fight could explain the low increase in the testosterone level, as its rise is mainly connected with the power aspect of workout [13]. Numerous papers have underlined the impact that testosterone has on the athletes' psychological reactions—reduction of fear and stimulation of aggression and motivation [17, 18].

The blood cortisol level is a good indicator of a person's adaptation to exercise, but it has been noted to be lower in male combat sports athletes than in a control male group [19]. Its secretion by adrenal glands is controlled by the hypothalamic-pituitary-adrenal axis and depends on the intensity, duration, and type of exercise [1]. The increase of the cortisol level during fight helps mobilize the energy reserves [20]. A rise in the blood cortisol level during short, repeated supramaximal exercise has already been reported [1, 21]. Our study confirms such an increase during a fight in all the combat sports analyzed. However, in the sumo, judo, and wrestling competitors, the postfight cortisol level was markedly lower than in the other disciplines, a finding not easy to interpret. It is highly unlikely that the exercise load was actually lower in JWS competition than in karate or taekwondo. Moreover, one should bear in mind that exercise-induced cortisol secretion displays high intersubject variability which depends on resistance to stress, degree of training, and exhaustion [22].

Sports medicine specialists often use the anabolic/catabolic index which is the testosterone to cortisol ratio (*T*/*C*) and is often employed as an index of overtraining and adaptation to exercise, modified by anabolic and catabolic metabolism and even by psychophysical stress and exhaustion [23]. Some authors [22] consider the ratio of the testosterone to cortisol resting levels to be an indicator of social aggression. The index is subject to change during training and is also used to assess the degree of overtraining [23]. In this paper, the *T*/*C* indices were similar in all groups, which indicates a similar physical load in all combat sports analyzed.

An increase in the blood BDNF level in athletes during the fight is the novelty of this paper. The arousal, however, was independent of the sports discipline, which requires further research. BDNF is a major regulator of synaptic transmission and plasticity in adult synapses in many areas of the central nervous system [16], and physical exercise on a treadmill has been shown to increase its blood concentration in a manner dependent on the load and distance covered [24]. To the best of our knowledge, our study is the first to investigate the effect of a short but very intense exercise during a combat sport fight on BDNF concentration. We found a rise in the blood BDNF level following the fight, similar for all the combat sport disciplines studied. This indicates that not only lengthy, steady exercise (such as on a treadmill) but also relatively short activity combined with emotional arousal causes BDNF to rise. Bearing in mind the overall positive influence of BDNF on mental performance, our result suggests that any type of exercise should be beneficial in this respect. The relative input of the exercise itself and the fight-associated stress on the BDNF upregulation is currently unknown and deserves further studies.

To reiterate, an increase in blood adrenaline, noradrenaline, cortisol, testosterone, and BDNF levels was observed following fight in all the combat sport disciplines studied by us. These hormonal changes reflect the neuroendocrine adaptation to competition-related exercise as well as aggression level and cognitive functioning. Karate competition stood out as it produced the largest increase in the noradrenaline and testosterone levels. It seems likely that differences in the increase of noradrenaline level between the groups do not reflect the physical load of the exercise itself but rather the different emotional engagement/aggression levels in the respective disciplines. The latter claim finds strong support in the finding of a significantly higher baseline (prefight) noradrenaline level in the karate athletes than in the taekwondo ones. Therefore, in light of the above, the smallest increase of the testosterone level during taekwondo fight is especially interesting whether it is due to training adaptation or to inborn predispositions which can be specified at an early stage of training specialization. On the other hand, the increase of the blood cortisol level following competition was similar in all groups analyzed. Both testosterone and cortisol are final components of the hormonal axis. Salvador et al. [25] have described anticipatory cortisol responses to competition and suggested that they are connected with the psychological preparation for competition, self-confidence, and motivation for success. Litwic-Kamińska [26] suggests that taekwondo and judo athletes from the Polish population are characterized by high resiliency, which makes them more resistant to stress. This could explain a significantly higher increase in the noradrenaline level in the karate group, but with regard to the testosterone level, such an association has been found in some athletes only.

The karate fighters studied here had the highest levels of hormones related to aggressive behavior; in contrast, earlier data on Polish sportsmen indicated that karate athletes were the least aggressive group compared to boxers, ju-jitsu fighters, and nontraining subjects [27]. Another study [28] showed that Polish wrestlers were more aggressive than karate fighters. It seems that in karate, the high level of hormones connected with aggression does not reflect the everyday behavior of the athletes but rather serves the situational, competitive aggression required for the stressful fight. Other data [29] also point out that the anger level in karate sportsmen is connected with the state rather than a trait when compared to nonathletes.

## 5. Practical Applications

This study is the first one to compare the hormonal response to competition in different combat sports. However, further studies are necessary to draw binding conclusions on the contribution of the emotional load of specific combat sport disciplines to the hormonal response. This applies to the physical and mental load and the aggression level as well. Further studies can lead to practical conclusions regarding the psychological training of combat sport athletes.

## 6. Conclusion

The relatively short bout of exercise in combat sports evokes an enormous activation of the sympathetic nervous system and the hypothalamic-pituitary-adrenal axis manifested by a major increase of plasma catecholamine, cortisol, and testosterone levels. The greatest increase of the bloodstream levels of noradrenaline concentration in karate could indicate a higher level of aggression in karate. However, in light of earlier studies indicating a low aggression level in karate, it is more likely to reflect the substantial contribution of stress associated with the acute struggle. Thus, the present study indicates that the hormonal effect of a sport fight is a combination of the effect of the physical exercise itself and the emotional arousal connected with combat performance.

## Figures and Tables

**Table 1:** Characteristics of training and competition demands in distinguished groups of combat sports.

	Technical characteristic	Energy demands	Contact with opponent
Karate group (K)	Power character: in kata marking techniques with no resistance	Depends on federation, in kumite (fight with opponent) mainly 3-4 min Mixed intensity in nonfighting forms	Limited in most federations No contact in kata (demonstration forms) and tests
Taekwondo group (T)	Power character: mainly kicking techniques Special techniques and strength tests (ITF Federation) and in WTF Federation special fitness competitions	WTF—high intensity, 3 × 2 min ITF—high intensity, 2 × 2 min Mixed intensity in nonfighting forms	Limited, possible use of protectors (in WTF Federation) Light contact in ITF No contact in kata (demonstration forms) and tests
Judo, wrestling, and sumo group (JWS)	Dynamic and static strength character: locks, holds, sweeps, lifting, throwing, pushing, & pulling	Judo—high intensity, duration 4 min Wrestling—high intensity, duration 3 × 2 min Sumo—high intensity, time not limited	Direct physical impact, highly stressful, high risk of direct injury

**Table 2:** Study subject characteristics (mean values ± SE).

	K group *n* = 24	T group *n* = 23	JWS group *n* = 81
Age (years)	21.9 ± 1.4	20.3 ± 0.5	20.2 ± 0.5
Body weight (kg)	75.6 ± 2.2	71.6 ± 2.3	78.3 ± 1.8
Height (m)	1.78 ± 1.1	1.78 ± 1.9	1.78 ± 0.9
Body mass index (kg/m^2^)	23.7 ± 0.5	22.3 ± 0.5	24.5 ± 0.4
Training experience (years)	10.3 ± 0.5	11.9 ± 1.3	7.9 ± 0.5

**Table 3:** Changes of blood adrenaline, noradrenaline, cortisol, testosterone, and BDNF levels before and after the fight in karate (group K), taekwondo (group T), and in judo, sumo, and wrestling (group JSW).

		Adrenaline (ng/ml)	Noradrenaline (ng/ml)	Cortisol (ng/ml)	Testosterone (ng/ml)	BDNF (pg/ml)
Group K *n* = 25	Before	^†^(*p* = 0.04)	^†^(*p* = 0.008)			
		2.7 ± 0.2	17.4 ± 1.9	114.5 ± 16.2	4.3 ± 0.4	217.1 ± 24.1
	After	^†^(*p* = 0.006)	†(*p* = 0.002)			
		6.3 ± 07^∗^	36.5 ± 4.8^∗^	209.1 ± 24.2^∗^	5.6 ± 0.5^∗^	431.3 ± 63.8^∗^
	*Δ*	3.6 ± 0.7	19.1 ± 3.6	94.6 ± 20.2	1.3 ± 0.16	214.2 ± 71.4
Group T *n* = 23	Before		^¢^(*p* < 0.001)			
		2.2 ± 0.3	9.5 ± 0.8	106.3 ± 10.9	3.7 ± 0.5	231.4 ± 23.6
	After		^¢^(*p* < 0.001)	^¢^(*p* = 0.014)		
		3.8 ± 0.4^∗^	14.6 ± 1.3^∗^	202.4 ± 19.8^∗^	4.5 ± 0.5^∗^	417.0 ± 56.8^∗^
	*Δ*	1.6 ± 0.24	5.0 ± 0.7	96.1 ± 17.2	0.8 ± 0.13	185.6 ± 84.3
Group JSW *n* = 81	Before				^#^(*p* = 0.013)	
		2.7 ± 0.3	15.2 ± 0.7	90.0 ± 5.3	3.2 ± 0.2	228.0 ± 31.5
	After	^#^(*p* = 0.007)		^#^(*p* = 0.024)		
		4.90 ± 0.5^∗^	23.6 ± 1.5^∗^	152.4 ± 6.5^∗^	4.6 ± 0.2^∗^	453.2 ± 86.2^∗^
	*Δ*	2.7 ± 0.4	8.4 ± 1.2	61.4 ± 5.2	1.4 ± 0.2	225.2 ± 79.4

Data are presented as the mean values ± SE. ∗ denotes significant differences between pre- and postfight values. ^∗^*p* < 0.001. † denotes differences between group K and group T. # denotes differences between group K and group JSW. ¢ denotes differences between group T and group JSW.

**Table 4:** Resting blood testosterone to cortisol ratio in the karate taekwondo group and in the wrestling, sumo, and judo group.

Group K	Group T	Group JSW
0.04 ± 0.01	0.03 ± 0.02	0.035 ± 0.02

Data are presented as the mean values ± SE.

## Data Availability

The data used to support the findings of this study are available from the corresponding author upon request.
